# Aneurysmal subarachnoid haemorrhage (aSAH): Five consecutive years' experience of Fars province, Iran

**DOI:** 10.1371/journal.pone.0189005

**Published:** 2017-11-30

**Authors:** Abdolkarim Rahmanian, Mohammad Jamali, Kamran Bagheri Lankarani, Sulmaz Ghahramani

**Affiliations:** 1 Department of Neurosurgery, Shiraz University of Medical Sciences, Shiraz, Iran; 2 Health Policy Research Center, Institute of Health, Shiraz University of Medical Sciences, Shiraz, Iran; Stellenbosch University Faculty of Medicine and Health Sciences, SOUTH AFRICA

## Abstract

**Objective:**

Subarachnoid haemorrhage (SAH), caused by the rupture of intracranial aneurysms, is a devastating event with high rates of morbidity and mortality. Aneurysmal subarachnoid haemorrhage (aSAH) plays a critical role in the potential loss of life as its sufferers are usually of a young age. We aimed to investigate the incidence of aSAH along with the patients’ characteristics over five consecutive years in Fars, a large province located in Southern Iran.

**Methods:**

In this prospective study, anonymous data of all patients diagnosed with aSAH in Fars province were collected after patient admission and surgery. Data from the last national census in 2011 were used to calculate the incidence. The data were analysed using SPSS software version 18 using independent sample t test, chi square test and ANOVA. The significance level was set at 0.05.

**Results:**

The number of aSAH cases identified in Fars, Iran, each year varied between 78 (2011) and 98 (2015) for a total of 421 aSAH cases within the 5-year study period. The annual aSAH incidence estimates showed no differences and were 1.65 [95% confidence interval (CI): 1.58–1.72], 1.70 (95%CI: 1.68–1.72), 1.71 (95%CI: 1.63–1.78), 1.82 (95%CI: 1.74–1.9), and 2.05 (95%CI: 1.97–2.13) per 100,000 persons, respectively, for the five consecutive years from 21 March 2011 to 20 March 2016. Hypertension was the most common risk factor, and was found in 198 (48%) aSAH patients. Ninety-four (22.5%) patients had moderate hydrocephalus on admission. Middle cerebral artery and anterior communicating artery were the most common sites of aneurysms. On admission, 351 (83%) patients had a Glasgow Coma Scale score >7, 197 (47%) presented with Hunt and Hess score of 1, and 365 (87%) had a Fisher score of ≤3. Multiple aneurysms were found in 59 (14%) of the 421 cases and the most common risk factors in multiple aneurysms were hypertension in 30 (51%) and smoking in 26 (44%) cases. Survival data were available only on patients diagnosed in year 2015, and the six-month survival rate was 89.8%.

**Conclusions:**

This study revealed that although the incidence of aSAH remained stable, the survival of aSAH patients who reached the hospital alive and were operated on, improved in Shiraz (the six-month survival rate was 89.8% in year 2015). The incidence and survival study on aSAH in other geographic areas of Iran as a multi-centre study is recommended. There is a need to inform primary healthcare workers regarding the possibility of aSAH in a patient with signs of the sentinel headache.

## Introduction

It is known that intracranial aneurysms (IAs) are found in approximately 2% of adults without risk factors for subarachnoid haemorrhage (SAH) [1]. Most of these aneurysms are small (5–10 mm), and the annual risk of rupture is estimated to be approximately 0.7% [1].

Factors that increase the rupture risk of IAs include active smoking, larger size of the un-ruptured aneurysm, and younger age [2]. Hypertension (HTN), cigarette smoking, family history of cerebrovascular disease, female sex, and postmenopausal state in female patients are significant risk factors for multiple aneurysms [3].

The incidence is comparable in most parts of the world (approximately 9 per 100,000 person-years) except for Japan and Finland where the incidence is higher and in South and Central America where the incidence is lower [4]. Not only have variations in the incidence of aSAH been observed among countries but also regional variation has been reported within countries, as was indicated in the Norwegian study, where the incidence increased from the south to the north [5].

In the Fars province, located in the Southern Iran, in a study carried out from September 2002 to March 2006 only 68 cases were identified during the four-year study period, which corresponds to an incidence estimate of <1/100,000 annually[6]. Another study from the same region, however, reported the incidence of aSAH to be 1.31/100,000 persons during a one-year period from March 2008 to March 2009 [7].

The objectives of our study were to 1) characterize the Iranian aSAH patient population and 2) re-assess the aSAH incidence in Fars, Iran to find out if there have been changes over the years. The findings of our study are crucial for better regional- and national-level planning and resource allocation of health care. Prospective data collection methods were used due to the sudden onset (unpredictable nature) of aSAH.

## Material and methods

The present study was approved by the Ethics Committee of the Shiraz University of Medical Sciences, Shiraz, Iran. Written informed consent was obtained from all patients diagnosed with aSAH after their admission to the hospital and after surgery, to use the anonymised clinical data for research purposes. If the patient was unconscious, the consent was obtained from the patient’s closest relative or from the person accompanying the patient to the hospital.

### Study population

This prospective study was performed in the Shiraz University of Medical Sciences, which is the only referral centre for cerebrovascular surgery in Southern Iran. Neurovascular surgery has been practiced for over 35 years in one of the busiest referral hospitals in this city, the Namazi Hospital. Shiraz is the centre of Fars province, which is the largest province southeast of Iran and had a population of 4,596,658 according to the 2011 Iranian census and 4,851,274 according to the 2016 census (https://www.amar.org.ir/english) demonstrating a 5.5% increase in the population of Fars province during the 5-year period. All patients diagnosed with aSAH in the Fars province are referred to this centre through the Monitoring Centre for Medical Care (MCMC).

### aSAH incidence estimates

The incidence of SAH and aSAH was calculated using an estimate of the population size from the national statistics data from years 2011 and 2016 (the national census in Iran is performed every 5 years and data were available from 2011 and 2016) (https://www.amar.org.ir/english) as the denominator and the number of SAH or aSAH incidences as the numerator.

### Clinical data

In this study, we used data from the aSAH database, which has patient data from the time period of 2011 to 2015 on 421 aSAH patients. Clinical data on the aSAH patients for the database were collected anonymously after obtaining written informed consent from the patients with diagnosis of aSAH (or from the patient’s closest relative, or from the person accompanying the patient to the hospital if the patient was unconscious) after hospital admission and after surgery. These data were collected by a neurosurgeon in a Data Collection Form (S2 File) and then entered to SPSS software by a data manager. Data were checked for entry errors and inconsistencies by one of the researchers. In this process, researcher randomly selected the questionnaires and checked them for any error of data entry. Data on clinical outcomes after surgery were collected only for year 2015 aSAH patients during the patients’ 6-month follow-up clinic visit (n = 80). If there was no follow-up visit (n = 15), (three deceased during hospital course) the clinical outcome information was collected by phone.

SAH was defined as any condition, in which blood enters the subarachnoid space including: idiopathic, infections, toxins, vascular, hematologic and neoplasm causes [8].

The diagnosis of SAH was based on an unenhanced brain-computed tomography (CT) scan. Lumbar puncture was performed to rule out xanthochromia in the case of a normal brain CT [8].

In patients suspected of having aSAH, brain CT angiography (CTA) was performed to confirm the diagnosis of IA. If the brain CTA was inconclusive, four-vessel angiography was performed within 7–10 days [9].

All clinical data (available in the Supplementary Data file, S1 File) for IAs and multiple IAs including the patients’ characteristics [record number, place of residency (province, city), phone number, month and year of admission, discharge and operation], other data including: demographic information (age, sex), co-morbidities [diabetes mellitus (DM), ischemic heart disease (IHD)], risk factors for aSAH (HTN, smoking) and pre-surgery characteristics (hydrocephalus, shunt before surgery, side and site of aneurysm and number of aneurysms) including pre-surgery scores of severity were gathered prospectively into the Data Collection Form from the medical records.

Pre-surgery scores of severity included Glasgow Coma Scale (GCS) score [10], the Fisher score [11], and the Hunt and Hess score [12]. Hunt and Hess scores change between 1 to 5 which corresponds with grade, 1 = asymptomatic or minimal headache and slight nuchal rigidity; 2 = moderate to severe headache, nuchal rigidity, and no neurological deficit other than cranial nerve palsy; 3 = drowsiness, confusion, or mild focal deficit; 4 = stupor, moderate to severe hemiparesis, possibly early decerebrate rigidity, and vegetative disturbances; and 5 = deep coma, decerebrate rigidity, moribund appearance [12].

Fisher score is graded according to patients' CT scan changes between 1 to 4, and includes 1 = no blood detected; 2 = a diffuse disposition or thin layer with all vertical layers of blood (interhemispheric fissure, insular cistern, ambient cistern), 1 mm thick; 3 = localized clots and/or vertical layers of blood ≥1 mm in thickness; and 4 = diffuse or no subarachnoid blood, but with intracerebral or intraventricular blood [11].

Information on risk factors (HTN, smoking) and co-morbidities (DM and IHD) were provided by the patient, or patient’s closest relative or the person accompanying the patient to the hospital, if the patient was unconscious, or the information was extracted from the medical records. Patient was considered hypertensive if the patient (or patient’s closest relative or the person accompanying the patient to the hospital, if the patient was unconscious) stated that he/she had positive history of HTN or was on antihypertensive medication. Patients with no history of HTN, but who had high blood pressure at the time of admission, were not considered hypertensive.

If the patient (or patient’s closest relative or the person accompanying the patient to the hospital, if the patient was unconscious) stated to have positive history of DM, or being on hypoglycemic medication, the patient was considered to have DM. A new diagnosis of DM was recorded if the patient had one of the following findings during the hospital stay: fasting plasma glucose ≥ 126 mg/dL, 2-hours plasma glucose ≥ 200 mg/dL, or HbA1C ≥ 6.5%.

For this study, both current and former smokers were considered smokers. Smoking history was obtained from the patient (or from patient’s closest relative or the person accompanying the patient to the hospital, if the patient was unconscious). Current smokers were defined as patients who had smoked ≥20 cigarettes/day for 1 year (1 pack-year) or the equivalent, and former smokers as any regular smokers (at least 1 pack-year) who had not smoked a cigarette for 3 months.

The patient was considered to have IHD, if it was stated by the patient (or patient’s closest relative or the person accompanying the patient to the hospital, if the patient was unconscious) that he/she had positive history of primary cardiac arrest, angina pectoris, myocardial infarction, heart failure and arrhythmia due to IHD, or the diagnosis was found in the medical records.

### Statistical analysis

Data were analysed using descriptive and analytical methods on the SPSS software version 18.0 (SPSS Inc., Chicago, IL, USA). We used the chi-square test for the comparison of qualitative variables, and the independent sample t-test and ANOVA for continuous variables. The significance level was set at 0.05.

## Results

### Incidence estimates for SAH and aSAH in Fars province, Iran

We obtained information on all SAH cases from the Namazi hospital information system associated with the Shiraz University of Medical Sciences and identified a total of 731 SAH patients during the 5-year period 2011–2015 (Table 1). The number of SAH patients diagnosed each year, varied from 143 in 2014 to 155 in 2015 (Table 1). Among the SAH patients, there were a total of 421 patients, who were diagnosed with an IA during the 5-year period 2011–2015, and were, therefore, classified as aSAH cases (Table 1). The incidences of SAH and aSAH showed only slight year-to-year variation, which was not significant (p = 0.67 and p = 0.51, respectively).

**Table 1:** Incidence estimates for SAH and aSAH in 2011–2015 in Fars, Iran.

Year	Number of SAH cases per year	Number of aSAH cases per year	Incidence estimates for SAH per 100,000 (95%CI)^a^	Incidence estimates for aSAH per 100,000 (95%CI)
2011	144	78	3.13 (3.04–3.22)	1.65 (1.58–1.72)
2012	145	79	3.12 (3.03–3.21)	1.70 (1.68–1.72)
2013	144	80	3.07 (2.98–3.16)	1.71 (1.63–1.78)
2014	143	86	3.02 (2.93–3.11)	1.82 (1.74–1.89)
2015	155	98	3.24 (3.15–3.33)	2.05 (1.97–2.13)
All 5 years combined	731	421	3.11 (3.02–3.20)	1.79 (1.74–1.84)

^a^The incidence of SAH and aSAH was calculated using an estimate of the population size from the national statistics data from years 2011 and 2016 (the national census in Iran is performed every 5 years and data were available from 2011 and 2016) (https://www.amar.org.ir/english) as the denominator and the number of SAH or aSAH incidences as the numerator. 95% Confidence intervals are also provided for estimates.

SAH, subarachnoid haemorrhage; aSAH, aneurysmal SAH.

### Characteristics of aSAH patients

The mean age of the 421 aSAH patients was 49.8± 1.5 years (Table 2). The mean age of the aSAH patients did not vary in the different years (p = 0.06). The female-to-male ratio was 1.15. There was no difference in the proportion of male patients (p = 0.6) in different years. Multiple aneurysms were present in 59 (14%) of the 421 cases.

**Table 2:** Characteristics, risk factors and comorbidities of aSAH patients.

Year	Age Mean± SD (years)	Male n (%)	Hypertension n (%)	Smoking^a^ n (%)	Diabetes mellitus n (%)	Ischemic Heart Disease ^b^ n (%)
2011	50± 1.4	38 (48.7)	38 (48.7)	27 (34.6)	3 (4)	9 (11.5)
2012	50± 1.6	33 (41.8)	32 (40.5)	19 (24)	6 (8)	22 (28)
2013	50.7± 1.5	38 (47.5)	41 (51.2)	19 (24)	17 (10)	41 (16.2)
2014	45.9± 1.6	45 (52.3)	34 (39.5)	17 (20)	5 (6)	9 (10.5)
2015	52.3±1.3	41 (41.8)	53 (54.1)	41 (42)	9 (9)	18 (18.4)
Total	49.8± 1.5	195 (46.3)	198 (47.1)	123 (29)	31 (7.1)	71 (16.8)

^a^Defined as current or former smoker.

^b^Includes: Primary cardiac arrest, angina pectoris, myocardial infarction, heart failure and arrhythmia due to IHD.

Individual level data are provided in the Supplementary Data File (S1 File).

### Comorbidities of aSAH patients

HTN, the most prevalent co-morbidity, was noted in 198 patients (47%). Smoking was seen in 123(9%) (Table 2).

Statistically, there were no significant differences in the percentage of DM (p = 0.5), or HTN (p = 0.2) among the aSAH patients diagnosed in different years, but significantly more patients reported smoking in 2015 than in the previous four years (p = 0. 006; Table 2).

### Comparison of patients with single and multiple IAs

There was no statistically significant difference between the mean age of multiple IA patients and that of single IA patients (single: 49.4 years; multiple: 52.5 years; p = 0.06). The female-to-male ratio among patients with multiple IAs was 1.03, whereas it was 1.18 among single IA patients.

Among the patients with multiple IAs, 26 (44.1%) were smokers, and 30 (50.8%) had HTN. Smoking was significantly more prevalent in patients with multiple than single IAs (p = 0.007). Co-morbidities and risk factors of aSAH patients, for single and multiple IAs separately are presented in Table 3.

**Table 3:** Comparison of the aSAH risk factors and comorbidities between aSAH patients with single and multiple intracranial aneurysms.

	Single n (%)	Multiple n (%)	p
HTN	168 (46.4)	30 (50.8)	0.5
Smoking	97 (26.8)	26 (44.1)	0.007
DM	27 (93.2)	4 (6.8)	0.8
IHD	62 (17.1)	9 (15.3)	0.7

HTN, hypertension; DM, Diabetes Mellitus; IHD, Ischemic Heart Disease.

Individual level data are provided in the Supplementary Data File(S1 File).

### Location and other features of IAs

Anterior communicating artery (ACOM) and middle cerebral artery (MCA) were the most common sites of IA; each was present in 128 (30.4%) aSAH cases (Table 4). A total of 147 (35%) patients had moderate or severe hydrocephalus on admission (Table 4).

**Table 4:** Location and other features of IAs.

Clinical feature		2011 n (%)	2012 n (%)	2013 n (%)	2014 n (%)	2015 n (%)	Total n (%)
Shunt before surgery		9 (9.2)	7 (8.1)	5 (6.2)	10 (12.2)	9 (11.5)	40 (9.5)
Hydrocephalus before surgery	Mild	18 (23)	10 (12.7)	16 (20.5)	15 (17.6)	18 (18.4)	77 (18.3)
	Moderate	9 (11.5)	22 (27.8)	14 (17.9)	21 (24.7)	28 (28.6)	95 (22.6)
	Severe	16 (20.5)	12 (15.2)	6 (7.7)	6 (7.1)	12(12.2)	52 (12.4)
Multiple IAs [≥ 2]		18 (23.1)	13 (16.5)	6 (7.5)	9 (10.5)	13 (13.3)	59 (14)
IA location^a^	MCA	31 (39.7)	21 (26.6)	29 (36.2)	23 (26.7)	39 (39.8)	128 (30.4)
	ACOM	26 (33.3)	23 (29.1)	17(21.2)	19(22.1)	28(28.6)	128(30.4)
	Other	21 (26.9)	35 (44.3)	34 (42.5)	44 (51.2)	31 (31.6)	165 (39.2)

^a^For cases with multiple aneurysms, site of the first IA is presented.

MCA, Middle Cerebral Artery; ACOM, Anterior Communicating Artery.

Individual level data are provided in the Supplementary Data File (S1 File).

On admission, 351 (83%) patients had GCS >7, and 294 (70%) presented with HH score of 1 (47%) or 2 (23%) and 365 (87%) had a Fisher score ≤3 (Table 5).

**Table 5:** Pre-surgery scores of severity.

Scoring System	Score	Number of patients N (%)
GCS	< = 7	70 (16.6)
	>7	351 (83.4)
Hunt and Hess	1	197 (46.8)
	2	97 (23)
	3	17 (4)
	4	55 (13.1)
	5	55 (13.1)
Fisher score	1	107 (25.2)
	2	56 (13.3)
	3	202 (48)
	4	56 (13.3)

GCS, Glasgow Coma Score

Individual level data are provided in the Supplementary Data File (S1 File).

The medians of the duration of the surgery and bleeding volume were 60 (in 2011) and 50 (in 2012) minutes, and 150 cc (in 2011) and 150 cc (in 2012), respectively.

In year 2015, the 6-month survival rate was 89.8%; only 10 (10.2%) patients died within six months of surgery.

## Discussion

The incidence of aSAH remained stable in Southern Iran from March 2011 to March 2015, and overall incidence of all 5 years was 1.79 per 100,000. Rakei et al. reported on the incidence of aSAH in this geographical area from 2002 to 2006 in 68 patients [6]. However, in the present study carried out 5 years later than the Rakei et al. study, the incidence was far higher, probably due to the implementation of the neurovascular sub-specialty programme in 2007, which has resulted in better diagnosis of these cases. Besides, the cost of services is now more affordable for patients, as these hospitals are affiliated with the Shiraz University of Medical Sciences. In addition, due to the complexity of the management of aSAH, surgeons in private services prefer to refer the patients to this referral centre.

A review of the literature revealed wide variations in the incidence of aSAH among different populations. The incidence was reportedly quite high in Finland and Japan [13]. In other areas, the incidence was not as high [14], even in countries with the same climatic conditions (for example Iceland) [15]. In the USA and Australia, the incidence estimates were 21.8/100,000 and 9.4/100,000 persons, respectively; however, the incidence of aSAH was over-estimated in Australia because of the inclusion of all causes of spontaneous SAH [16, 17]. The lower incidence in Fars may be due to referral system failures, poor diagnostic capabilities, and poor relationship between different medical specialities or death before receiving medical attention. Another possibility could be differences in the genetic susceptibility of the population to a aSAH, which was mentioned by Ohkuma et al., who stated that the high incidence of aSAH in Finland and Japan could be due to genetic factors [18]. The genetics of IAs is, however, complex and involves multiple genetic factors and gene-environment interactions [19].

In an incidence trend study in Japan, the trend of incidence remained stable during a 19-year period; meanwhile, the case fatality rate of the studied patients showed improvement [13]. Therefore, decreasing the case fatality of aSAH may be due to better management of the patients [20], the evaluation of aSAH patients’ survival besides the trend of incidence is important for the evaluation of its management.

The incidence rate of aSAH was slightly higher in women than men in the current study, but the percentage of aSAH in the previous studies was higher in women than men [17,18]. Therefore, more multicentre studies are needed to explore the risk factors for aSAH in Iran. Also, the possibility of under-reporting (especially in women) should be kept in mind. To decrease under-reporting, diagnosis and referral protocols should be prepared, and the trend of incidence should be followed and reported. Autopsy studies to confirm the cause of death as well as supervised and correct registration of death codes may help to correct the possible causes of under-reporting.

The most prevalent aSAH risk factors [21,22], were HTN and smoking. Among the 10 deceased patients of year 2015, six were smokers and four had HTN. The reported prevalence of multiple IAs in aSAH patients in other countries was 27–30% [3, 21] but in Shiraz it was only 14%.

Although smoking, HTN, and female sex are known to be strong risk factors for multiple IAs in other populations [23], in the Iranian population, only smoking was more common among patients with multiple than single IAs. Further studies on the risk factors of multiple aneurysms are strongly recommended [3].

In a previous report from our centre in Iran MCA (33.2%) was the most common site of IA [24], but according to the results of this study, ACOM and MCA were equally common sites.

As a result of the sub-specialty programme in Shiraz, diagnostic and surgical outcomes have improved. The six-month survival rate of the aSAH patients given in the previous report of this centre based on follow up of 160 patients was 86.2% [24]; however, in this study, the six-month survival indicated an improvement in the year 2015 (89.8%). In a meta-analysis on 8,739 patients the improvement of the outcome of aSAH surgery since 1965 to July 2007, the best survival was reported at 92% (after one year)[25]. This meta-analysis did not include data of Middle East but it seems that our survival numbers are surprisingly high to numbers from other countries.

Our study has several limitations, the main one being the fact that it was a single-center study and that it relied on patient questionnaires for most of the risk factor information. Furthermore, our study did not include data of outcomes of patients for the first four years of the study, and no data on several aSAH-associated risk factors (e.g. alcohol use and cerebrovascular diseases) were collected. It is also possible that several aSAH patients died before reaching the hospital and were, therefore, not included in the study. These factors might have caused a selection bias and the results might not be representative of all aSAH patients in Iran.

This study helps us to gain an overview on aSAH in the Middle East. It is likely that we underestimated the aSAH incidence because most patients who presented with the rupture of aSAH may not have reached the hospital, as stated also in the Minnesota study [26]. In a meta-analysis of a study based on 18 populations, it was found that before a patient with a ruptured aSAH receives medical attention, the combined overall estimated risk of sudden death is 12.4% and 44.7% for aSAH and posterior circulation aneurysms, respectively [27, 28]. Pre-hospital death rate should, therefore, be evaluated in our region in Iran. For better comparison, a multi-centre study of the incidence or incidence trends of aSAH in other geographic areas of Iran is recommended.

## Conclusions

This study revealed that although the incidence of aSAH remained stable, and the survival of aSAH patients who reached the hospital alive and were operated on, improved in Shiraz (the six-month survival rate was 89.8% in year 2015). The incidence and survival study on aSAH in other geographic areas of Iran as a multi-centre study is recommended. There is a need to inform primary healthcare workers regarding the possibility of aSAH in a patient with signs of the sentinel headache.

## Supporting information

S1 File Individual level data. (XLSX)

S2 File Data collection form. (DOCX)
