# Supplementary material for: Aneurysmal subarachnoid haemorrhage (aSAH): Five consecutive years' experience of Fars province, Iran
Source: PLoS One. 2017 Nov 30;12(11):e0189005. doi: 10.1371/journal.pone.0189005 (PMC5708774; doi:10.1371/journal.pone.0189005)
Supplement: S2 File — (DOCX) [file pone.0189005.s002.docx]

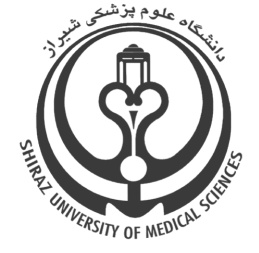

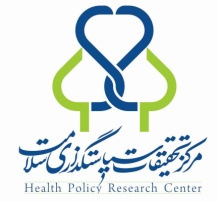


**In the name of GOD**

**Data collecting form for aneurismal subarachnoid hemorrhage- Namazi hospital**

Date:

ID number of form:

**Patient characteristics:**

1. Patient record code: 2- phone number (at least one):
2. Province and City of residency:
3. Age (year):
4. Sex: male□ female□
5. Month and year of admission: 7- Month and year for aSAH:

8- Month and year of surgery: 9- Month and year of discharge:

10- GCS: 11-Hunt &Hess: 12-FISHER score:

13- Hydrocephalus before surgery: No□ mild□ moderate□ severe□

14- Shunt before surgery (VP): yes□ No□

15- Co morbidity and risk factors (defined below in last page):

- Ischemic heart disease: yes□ No□
- diabetes mellitus: yes□ No□
- smoking: yes□ No□
- Hypertension: yes□ No□

**Surgery data:**

16- Number of aneurysm/s:

17- Site of aneurysm/s: 1- 2- 3- 4- 5- 6-

18- Size of aneurysm/s: 1- 2- 3- 4- 5- 6-

19- Size of clip/s: 1- 2- 3- 4-

5- 6-

20- Type of clip/s: 1- 2- 3- 4-

5- 6-

21-Rupture during surgery: yes□ No□

22- Amount of bleeding (CC):

23- Using temporary clip during surgery: yes□ No□

24-Duration of surgery (min):

25-Type of surgery:

Further Comments about patient:

**Definitions**:

1. HTN: positive history of HTN or being on antihypertensive medication stated by patient (or his/her closest relative in case of patient unconsciousness). Patients with no history of HTN which have high blood pressure at the time of admission are not considering as HTNve.
2. Diabetes: positive history of DM, or being on hypoglycemic medication stated by patient (or his/ her closest relative in case of patient unconsciousness). New DM patients with one of the following findings during hospital admission: Fasting plasma glucose ≥ 126 mg/dL, 2-hours Plasma Glucose ≥ 200 mg/dL, HbA1C ≥ 6.5%.
3. Smoking: (both current and former or ex-smoker): positive history of smoking stated by patient (or his/her closest relative in case of patient unconsciousness): Current smokers were defined as patient who had smoked at least 20 cigarettes per day for 1 year (1 pack year) or the equivalent and former smokers as any reformed regular smoker (at least 1 pack year) who had not smoked a cigarette for 3 months.
4. IHD including: Primary Cardiac Arrest, angina pectoris, myocardial infarction, heart failure and arrhythmia due to IHD stated by patient (or patient’s closest relative or the person accompanying the patient to the hospital If the patient was unconscious) or extracted from medical records.
